# Clinical response to EPA supplementation in patients with major depressive disorder is associated with higher plasma concentrations of pro-resolving lipid mediators

**DOI:** 10.1038/s41386-022-01527-7

**Published:** 2023-01-12

**Authors:** Stefania Lamon-Fava, Minying Liu, Boadie W. Dunlop, Becky Kinkead, Pamela J. Schettler, Jennifer C. Felger, Thomas R. Ziegler, Maurizio Fava, David Mischoulon, Mark Hyman Rapaport

**Affiliations:** 1 Jean Mayer USDA Human Nutrition Research Center on Aging at Tufts University, Boston, MA USA; 2 Department of Psychiatry and Behavioral Sciences, Emory University, Atlanta, GA USA; 3 Huntsman Mental Health Institute, Department of Psychiatry, University of Utah, Salt Lake City, UT USA; 4 Department of Medicine, Emory University, Atlanta, GA USA; 5 Department of Psychiatry, Massachusetts General Hospital, Boston, MA USA

**Keywords:** Depression, Predictive markers

## Abstract

Chronic inflammation has been implicated in the pathophysiology of major depressive disorder (MDD). Activating the resolution of inflammation through ω-3 fatty acid supplementation may prove to be a successful therapeutic strategy for the treatment of MDD. Patients with MDD, body mass index >25 kg/m^2^, and plasma high-sensitivity C-reactive protein ≥3 μg/mL (*n* = 61) were enrolled in a 12-week randomized trial consisting of 4 parallel arms: EPA 1, 2, and 4 g/d, and placebo. The supplement contained EPA and DHA in a 3.9:1 ratio. Depression symptoms were assessed using the IDS-C30 scale. Plasma fatty acids and pro-resolving lipid mediators (SPMs) were measured in 42 study completers at baseline and at the end of treatment by liquid chromatography/mass spectrometry. The response rate (≥50% reduction in IDS-30 score) was higher in the 4 g/d EPA arm than placebo (Cohen *d* = 0.53). In the 4 g/d EPA arm, responders had significantly greater increases in 18-hydroxyeicosapentaenoic acid (18-HEPE) and 13-hydroxydocosahexaenoic acid (13-HDHA) than non-responders (*p* < 0.05). Within the 4 g/d EPA arm, the increase in 18-HEPE was significantly associated with reductions in plasma hs-CRP concentrations (*p* < 0.05) and IDS-C30 scores (*p* < 0.01). In summary, response rates were greater among patients with MDD randomized to EPA 4 g/d supplementation and in those who showed a greater ability to activate the synthesis of 18-HEPE. The inverse association of 18-HEPE with both systemic inflammation and symptoms of depression highlights the activation of the resolution of inflammation as a likely mechanism in the treatment of MDD with ω-3 fatty acid supplementation.

## Introduction

Chronic low-grade inflammation plays an important role in the pathogenesis of several non-communicable diseases, including those affecting the central nervous system. Symptoms of major depressive disorder (MDD) are frequently observed when the immune system is activated, such as during infections or in inflammatory autoimmune diseases or conditions associated with chronic inflammation [1–3]. Inflammation is characterized by an initial phase of immune cell activation and secretion of pro-inflammatory cytokines, prostaglandins (PG), and leukotrienes (LT), followed by the resolution phase of inflammation [4, 5]. The resolution phase is mediated by specialized pro-resolving lipid mediators (SPMs) derived in large part from the conversion of the ω-3 fatty acids eicosapentaenoic acid (EPA, 20:5 ω3), docosapentaenoic acid (DPA, 22:5 ω3) and docosahexaenoic acid (DHA, 22:6 ω3) via cyclooxygenase (COX) and lipoxygenase (LOX) enzymatic pathways [6]. SPMs include resolvins (Rv), protectins (P), and maresins (Mar) that act by binding to specific membrane receptors present on peripheral immune cells, but also on neurons and microglia [7–9]. The resolution phase of inflammation is characterized by efferocytosis of cell debris and apoptotic cells by specialized macrophages and by the counter-regulation of pro-inflammatory mediators to promote homeostasis [6].

Chronic inflammation is thought to result from an impaired resolution phase [6]. Several studies have shown dysregulation of SPMs in conditions associated with low-grade chronic inflammation, such as obesity and cardiovascular disease [10, 11]. Lower concentrations of SPMs have also been observed in the cerebrospinal fluid and the hippocampus of patients with neurodegenerative diseases compared to controls [8]. However, it is not known whether deficient concentrations of SPM result from lower dietary intakes of the ω-3 fatty acid precursors or from an impaired ability to activate the resolution machinery to produce SPM.

MDD is common among children and adults with obesity [12], and the rate of depression increases with BMI [13]. Obesity is characterized by low-grade chronic inflammation and is associated with lower SPM concentrations in adipose tissue [14, 15]. In mice, obesity and dietary intake of ω-3 fatty acids have opposite effects on MFSD2A2 (major facilitator superfamily domain-containing protein 2), a cell membrane transporter that mediates the translocation of DHA across the blood-brain barrier [16]. Therefore, it has been hypothesized that obesity may lead to reduced availability of ω-3 fatty acids in the brain and lower local SPM synthesis. However, leukocytes from individuals with obesity also display lower activity of 15-LOX, an enzyme involved in the synthesis of several SPMs [10]. We and others have shown that plasma and tissue concentrations of ω-3 fatty acids can be increased via supplementation in a dose-dependent fashion [17–19]. This has the potential to increase the substrate availability for SPM production [17–19].

Meta-analysis studies of randomized controlled trials examining the potential of ω-3 supplements to improve clinical depression have reported reduced depression severity with formulations containing pure EPA or greater than 60% EPA, but not with supplements containing pure DHA or greater than 60% DHA [20, 21]. EPA supplementation may be an effective therapeutic option for the sub-group of patients with MDD who are overweight or with obesity and have low-grade chronic inflammation [22]. However, the threshold for effective SPM synthesis following ω-3 supplementation is not known. In addition, in the current era of precision medicine, it is important to identify biomarkers that may predict therapeutic response and the mechanism(s) driving the response.

We had previously conducted a randomized, double-blind, placebo-controlled, parallel-arm study in patients with MDD to evaluate the clinical response to three different doses of EPA (1 g/d, 2 g/d, and 4 g/d) and the association between reduction in depression severity following EPA supplementation and changes in inflammation, as assessed by plasma high-sensitivity C reactive protein (hs-CRP) and peripheral blood monocyte mitogen-stimulated tumor necrosis factor α (TNFα) production [23]. The study showed better response rates and lower plasma hs-CRP levels with EPA 4 g/d than placebo. The current study is a secondary analysis of the EPA dose finding study and is aiming at assessing the biosignature of response to EPA supplementation in patients with MDD.

## Methods

### Study participants

The study design and participants’ characteristics have been previously described [23]. Briefly, eligible individuals were required to have non-psychotic MDD (diagnosed using the Mini International Interview version 7.0.2), a depression severity score ≥ 25 on the Inventory of Depressive Symptomatology-30 item (IDS-C30), a body mass index (BMI) > 25 kg/m^2^, and low-grade chronic inflammation (plasma hs-CRP, ≥ 3 µg/mL). Participants were recruited at two sites, Emory University (Atlanta, GA) and Massachusetts General Hospital (Boston, MA). Subjects with a high dietary intake of ω-3 fatty acids, as assessed by dietary history using Food Processor, or taking fish oil supplements were excluded from the study. Sixty-one subjects enrolled into the double-blind study and 45 completed it. Forty-two participants had baseline and week 12 plasma samples obtained for the assessment of SPMs.

### Study design

The study had a randomized, double-blind, placebo-controlled, parallel-arm design, consisting of four arms: (1) placebo, (2) EPA 1 g/d, (3) EPA 2 g/d, and (4) EPA 4 g/d [23]. The supplementation phase lasted 12 weeks. EPA was provided in capsules, each containing approximately 590 mg of EPA and 152 mg DHA in triglyceride form. Matched placebo capsules contained soybean oil (approximately 51% linoleic acid, 25% oleic acid, and 6% α-linolenic acid, but no EPA or DHA). Study participants were instructed to take eight capsules each day, four in the morning and four in the evening, with meals. Study subjects were asked to avoid taking non-steroidal anti-inflammatory drugs (NSAIDs) for at least 24 h before visits. The IDS-C30 scale is a validated clinician-rated measure which has been previously described [24]. Hs-CRP was measured by an immunoturbidometric method using reagents and calibrators from Sekisui. The study protocol was approved by the IRB at both institutions and is registered on ClinicalTrials.gov (NCT02553915). All subjects provided written informed consent.

### Fatty acid and SPM analysis

Fasting blood samples were collected in the morning in EDTA tubes at weeks 0 (baseline) and 12, centrifuged at 1000 *g* for 25 min at 4 °C, and plasma was immediately stored at −80 °C until analysis. All samples were batch-analyzed at the end of the study. For the analysis of plasma fatty acids, samples were hydrolyzed, and the fatty acid concentrations were assessed by ultrahigh-performance liquid chromatography/mass spectrometry as previously described [17, 25]. The concentration of individual fatty acids was validated against standard curves generated for each fatty acid. Fatty acid concentrations were then converted to molar percent of total plasma fatty acids (mol%). The plasma concentration of lipid mediators derived from EPA, DPA, DHA, and AA were assessed by liquid chromatography-mass spectrometry as previously described [17, 26, 27]. Briefly, 100 μl of plasma was spiked with 5 ng each of PGE_1_-d_4_, RvD2-d_5_, LTB_4_-d_4_, and 15S-hydoxyeicosatetraenoic acid-d_8_ (15S-HETE-d_8_) as internal standards for analyte recovery and quantitation. Lipid metabolites were extracted with C18 extraction columns and then subjected to LC-MS analysis. Data were collected with Analyst 1.6 software and quantitated using MultiQuant software (AB Sciex). All quantified lipid mediators were identified by comparison with authenticated standards (Cayman Chemicals, Ann Arbor. MI).

### Peripheral blood mononuclear cells

Peripheral blood mononuclear cells (PBMC) were isolated from fasting blood collected in sodium citrate Vacutainer Cell Preparation Tubes (Becton Dickinson, NJ). Cells were resuspended in cell culture medium (RPMI-1640, 10% fetal bovine serum, 100 U penicillin, 100 µg streptomycin) and plated in 12-well plates at the density of 2 × 10^6^ cell/well. Two wells were treated as control and two wells were treated with 10 ng/ml lipopolysaccharide (LPS). After 5 h incubation, cells were scraped and cell pellet and cell culture medium were separately collected after centrifugation. Concentrations of interleukin-6 (IL-6) and tumor necrosis factor α (TNF-α) in cell culture media were quantified using V-PLEX immunoassay kits from Meso Scale Diagnostics (MD).

### Statistical analyses

Statistical analyses were conducted in R (version 3.5.2). Variables with skewed distribution are presented as median and interquartile range (75 percentile—25 percentile). Response to treatment was defined as ≥ 50% reduction in IDS-C30 scores at week 12, relative to baseline. Differences between responders and non-responders were assessed by exact Wilcoxon–Mann–Whitney test. Partial Least-Squares Discriminant Analysis (PLS-DA) of the change in plasma lipid mediators was performed on normalized and scaled data using MetaboAnalyst 5.0 (https://www.metaboanalyst.ca), a web-based platform for comprehensive analysis of quantitative metabolomic data. Associations between depression scores, inflammation, and fatty acid and lipid mediator concentrations were assessed by Spearman’s rank-order correlation tests. A *p* value ≤ 0.05 was considered statistically significant.

## Results

The results of primary outcomes of the trial have been reported [23]. The study participants’ age, BMI, depression scores, and plasma hs-CRP concentrations at baseline in the four treatment arms are shown in Table 1. There were no significant differences in baseline characteristics among the four groups. Moreover, baseline plasma concentrations of EPA, DPA, DHA, and AA, and of their related lipid mediators, were similar among the four arms (Supplementary Table). Response to treatment occurred in 16 of the 35 subjects in the EPA treatment arms [5 of 13 (38%) in EPA 1 g/d, 4 of 11 (36%) in EPA 2 g/d, and 7 of 11 (64%) in EPA 4 g/d arm], and in 4 of the 10 subjects in the placebo arm (40%). At baseline, the amount of plasma EPA, DHA, and AA and the concentration of their related lipid mediators were similar between responders and non-responders (Table 2).

**Table 1:** Baseline characteristics of study participants by treatment group.

	Placebo (*n* = 10)	EPA 1 g/d (*n* = 13)	EPA 2 g/d (*n* = 11)	EPA 4 g/d (*n* = 11)
Men/women (*n*)	2/8	4/9	3/8	4/7
Age (y)	52 ± 13	41 ± 16	47 ± 15	43 ± 15
BMI (kg/m^2^)	38.8 (9.6)	33.0 (6.7)	34.3 (7.7)	35.7 (8.8)
Hs-CRP (µg/mL)	6.9 (4.5)	3.6 (8.3)	4.8 (1.2)	3.9 (4.7)
IDS-C30	36.6 ± 10.5	36.1 ± 7.4	31.4 ± 7.1	31.5 ± 5.0

Data presented as mean ± SD or median (IQR).

**Table 2:** Baseline plasma concentrations of EPA, DPA, DHA, AA and their derived lipid mediators in non-responders versus responders in the combined active treatment arms (EPA 1 g/d, 2 g/d, and 4 g/d).

	Non-responders (*n* = 19)	Responders (*n* = 16)	*P* value*
*EPA*			
EPA, mol%	0.42 (0.32)	0.43 (0.36)	0.85
5-HEPE, pg/mL	50 (29)	55 (53)	0.31
11-HEPE, pg/mL	43 (48)	49 (38)	0.70
12-HEPE, pg/mL	806 (2094)	588 (1838)	0.60
15S-HEPE, pg/mL	92 (62)	79 (87)	0.67
18-HEPE, pg/mL	47 (29)	47 (25)	0.88
*DPA*			
DPA, mol%	0.37 (0.17)	0.34 (0.26)	0.83
RvD5 _DPA_, pg/mL	15 (6)	14 (3)	0.73
*DHA*			
DHA, mol%	7.42 (4.59)	7.11 (3.40)	0.42
4-HDHA, pg/mL	60 (45)	56 (39)	0.57
7-HDHA, pg/mL	18 (14)	18 (17)	0.93
13-HDHA, pg/mL	14*5* (251)	165 (248)	0.60
14-HDHA, pg/mL	1810 (4012)	1580 (2254)	0.55
17-HDHA, pg/mL	30 (25)	31 (26)	0.82
RvD1, pg/mL	85 (103)	120 (121)	0.95
*AA*			
AA, mol%	10.11 (3.68)	10.52 (5.66)	0.80
5-HETE, pg/mL	355 (326)	445 (515)	0.40
11-HETE, pg/mL	1603 (1956)	1234 (2225)	0.67
12-HETE, pg/mL	9622 (13191)	9502 (15867)	0.99
15-HETE, pg/mL	1182 (689)	1306 (1268)	0.53
PGE_2_, pg/mL	1139 (1536)	965 (1904)	0.80
TXB_2_, pg/mL	2634 (3220)	1528 (4013)	0.72
LTB_4_, pg/mL	31 (17)	21 (29)	0.29

Median (IQR); *P value, Wilcoxon rank-sum test.

The changes in plasma EPA, DHA and AA, defined as the difference between week 12 and baseline, were similar in responders and non-responders within each treatment arm (Supplementary figure). A non-significant trend (*p* = 0.25) was observed for EPA, but not DHA, in the 4 g/d arm.

**Fig. 1:**
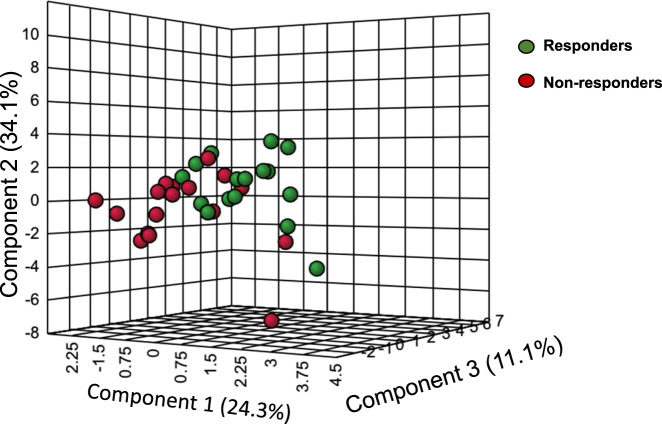
PLS-DA of changes in lipid mediators derived from EPA, DHA and AA in the combined treatment arms. Responders are shown in green dots and non-responders in red dots.

The PLS-DA score plot of the changes in lipid mediators derived from EPA, DHA, and AA in all subjects receiving active EPA supplementation indicated a partial separation of responders and non-responders (Fig. 1).

**Fig. 2:**
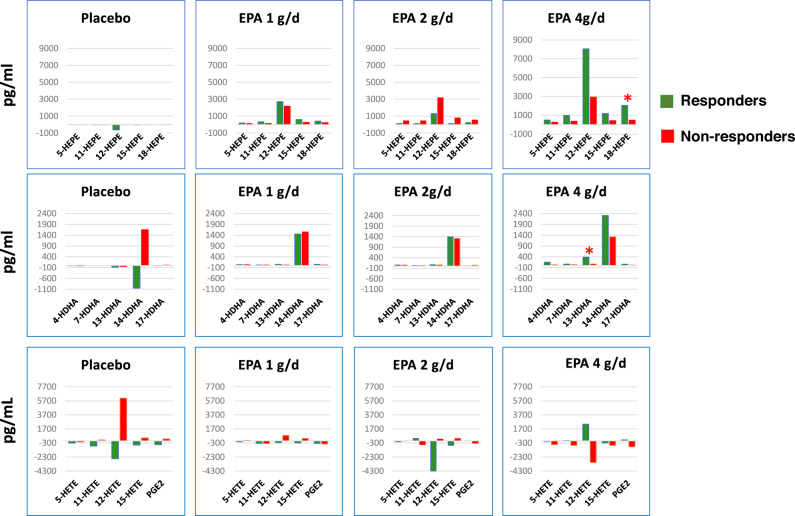
Median changes in lipid mediators in responders (green) and non-responders (red) by treatment group. Top row: EPA-derived lipid mediators; middle row: DHA-derived lipid mediators; and bottom row: AA-derived lipid mediators. *p < 0.05, responders versus non-responders in the same treatment arm.

Figure 2 shows the change in lipid mediators following treatment in responders and non-responders by treatment arm. The change in plasma 18-HEPE was significantly greater in responders than non-responders in the 4 g/d arm (*p* < 0.01), but a trend for greater increases in responders than non-responders was also observed for 11-HEPE and 15-HEPE (*p* = 0.067) (Fig. 2). In the 4 g/d EPA arm, the change in plasma DHA-derived 13-HDHA was also significantly greater in responders than non-responders (*p* < 0.05) (Fig. 2). There was a non-significant trend for a lowering of AA-derived lipid mediators in non-responders, and an increase in 12-HETE and PGE_2_ in responders in the EPA 4 g/d arm (Fig. 2). No significant differences between responders and non-responders were observed in the 1 and 2 g/d arms. When all EPA treatment groups were combined, changes in TXB_2_ were significantly higher (median [IQR], 431 [3,600] and −1250 [−2535] pg/mL, respectively; *p* = 0.04) and changes in 18-HEPE (594 [1597] and 361 [426] pg/mL; *p* = 0.067) and 13-HDHA (311 [402] and 51 [202] pg/mL; *p* = 0.067) were higher in responders than non-responders. RvE1 was not detected by our assay at baseline and end of treatment. However, RvE2 was detected at the end of treatment in four out of six responders and none of four non-responders, and RvE3 in five of six responders and one of four non-responders.

Within the 4 g/d EPA arm, reductions in hs-CRP were significantly correlated with reductions in IDS-C30 scores (*p* < 0.05) (Table 3). Increases in 18-HEPE, 15-HEPE, and 17-HDHA were significantly correlated with reductions in IDS-30 scores. In addition, increases in 18-HEPE and 15-HEPE were significantly correlated with reductions in hs-CRP.

**Table 3:** Spearman correlations among changes in variables from baseline to week 12 in the 4 g/d treatment group.

	IDS-C30	Hs-CRP	IL-6 (PBMC)	TNF (PBMC)	5-HEPE	11-HEPE	12-HEPE	15-HEPE	18-HEPE	4-HDHA	7-HDHA	14-HDHA	17-HDHA	5-HETE	11-HETE	12-HETE	15-HETE	PGE_2_	TXB_2_
IDS-C30	1	0.61*	0.11	0.17	−0.21	−0.57	−0.20	−0.71*	−0.80**	−0.27	−0.04	−0.15	−0.77*	−0.08	−0.17	−0.06	0.03	−0.40	−0.34
Hs-CRP		1	−0.10	0.37	−0.30	−0.50	−0.34	−0.68*	−0.73*	−0.11	−0.31	−0.15	−0.28	0.10	0.02	0.01	−0.11	−0.12	−0.06
IL-6 (PBMC)			1	0.21	18	0.09	−0.22	0.12	−0.04	−0.26	−0.30	−0.17	−0.13	0.07	0.04	−0.10	0.03	0.65	0.20
TNF (PBMC)				1	−0.73*	−0.60	−0.29	−0.64*	−0.07	−0.48	−0.75*	−0.05	−0.53	−0.39	−0.15	−0.03	−0.13	−0.41	0.58
5-HEPE					1	0.89**	0.65*	0.69*	0.32	0.89**	0.93***	0.47	0.87**	0.78**	0.62^	0.38	0.61*	−0.17	−0.37
11-HEPE						1	0.60	0.87**	0.55	0.79**	0.73*	0.46	0.79**	0.67*	0.67*	0.35	0.57	0.07	−0.07
12-HEPE							1	0.40	0.52	0.65*	0.75*	0.95***	0.89**	0.46	0.39	0.86**	0.60	−0.41	0.05
15-HEPE								1	0.57	0.51	0.54	0.23	0.51	0.37	0.29	0.11	0.18	0.00	−0.17
18-HEPE									1	0.33	0.23	0.43	0.39	−0.09	0.08	0.29	0.00	0.24	0.04
4-HDHA										1	0.83**	0.54	0.83**	0.79**	0.74*	0.40	0.68*	−0.38	−0.20
7-HDHA											1	0.56	0.92***	0.74*	0.50	0.47	0.60	−0.39	−0.48
14-HDHA												1	0.79**	0.42	0.43	0.95***	0.66*	0.32	0.24
17-HDHA													1	0.73*	0.66*	0.72*	0.77**	−0.24	−0.09
5-HETE														1	0.85**	0.34	0.86**	−0.17	−0.38
11-HETE															1	0.40	0.91	0.14	0.08
12-HETE																1	0.67	−0.23	0.34
15-HETE																	1	0.00	0.05
PGE_2_																		1	0.62*
TXB_2_																			1

*p < 0.05, **p < 0.01, ***p < 0.001.

## Discussion

EPA supplementation reduced clinical symptoms of depression, defined as achieving ≥ 50% reduction in IDS-C30 scores, in 64% of participants in the 4 g/d arm, relative to 40% in the placebo arm. At baseline, study participants who responded to treatment had plasma concentrations of EPA, DHA, DPA, AA and related lipid mediators similar to non-responders. However, after supplementation, there was a trend for greater increases in some of the lipid mediators derived from these fatty acids in responders versus non-responders, and differences were significant for 18-HEPE and 13-HDHA in the 4 g/d arm. To the best of our knowledge, this is the first clinical trial demonstrating a differential response to EPA supplementation in patients with MDD, with overall greater ability to synthesize EPA- and DHA-derived lipid mediators in responders than non-responders.

Chronic low-grade inflammation is a characteristic feature of metabolic syndrome, obesity, and cardiovascular disease, and has recently emerged as a contributing factor to diseases of the central nervous system, including MDD, Parkinson’s disease and Alzheimer’s disease [2, 28]. Impairment in the active process of inflammation resolution is thought to play a significant role in chronic inflammation, and several pre-clinical and clinical studies have demonstrated lower levels of SPM production associated with chronic inflammation. Mice on a high-fat diet had higher concentrations of pro-inflammatory lipid mediators and lower concentrations of SPMs in their adipose tissue, compared to control mice [14, 29]. In subjects with obesity, it has been reported that leukocytes had significantly lower levels of DHA-derived SPMs, relative to levels of the classic inflammatory mediators LTB4 and PGs, than subjects without obesity [10]. Lower SPM concentrations have also been observed in subjects with cardiovascular disease [11]. A healthy control group was not included in our study and therefore we do not know if the baseline plasma concentrations of ω-3 fatty acids and lipid mediators in our participants with MDD were lower than those of healthy age- and sex-matched subjects. At baseline, patients with MDD who responded to EPA supplementation had plasma concentrations of EPA and DHA and of their derived lipid mediators similar to non-responders, but responders showed a greater increase in EPA- and DHA-derived lipid mediators following supplementation than non-responders. Since responders and non-responders achieved overall similar plasma concentrations of EPA, DHA, and AA following supplementation, it is unlikely that the availability of precursors for the synthesis of SPMs played a significant role in response to supplementation.

Other mechanisms such as the activation of enzymatic pathways involved in SPM synthesis may account, at least in part, for the differences in response. Immune cells of subjects with obesity exhibit lower concentrations of 17-HDHA, a 15-LOX metabolite of DHA, but, when these cells were incubated with 17-HDHA, the production of SPMs was rescued [10]. These findings suggest a potential deficit in 15-LOX activity in subjects with obesity which may lead to lower synthesis of precursors and SPMs. In our study, other metabolites derived from other enzymatic pathways were also different between responders and non-responders. Specifically, 18-HEPE was one of the most important lipid mediators differentiating responders and non-responders. 18-HEPE is synthesized by both COX and cytochrome P450. It has been shown that COX-2 activation follows a biphasic pattern during inflammation: the first peak occurs during the early pro-inflammatory phase and is associated with increased PGE_2_ secretion and pro-inflammatory cytokine expression, and the second peak coincides with the resolution phase of inflammation and is characterized by increased PGD_2_ synthesis, reduction in neutrophil recruitment, and increase in macrophage mobilization from the inflammation site [30, 31]. In our study, TXB_2_, which is also synthesized in response to COX-2 activation, was significantly elevated after EPA supplementation in responders. Therefore, it may be hypothesized that the biphasic COX-2 activation is an important player in the resolution phase of inflammation and that both EPA- and AA-derived lipid mediators may be increased during resolution.

The results of our study are somewhat in contrast with those of a study conducted in 16 patients with MDD and supplemented with 1.6 g/d EPA and 0.8 g/d DHA for six weeks, where responders had higher mol% DHA, but not EPA mol%, in plasma phospholipids than non-responders [32]. It should be noted that in that study none of the responders but most of the non-responders had previously taken antidepressants and therefore the results may have been affected by patient characteristics related to treatment resistance. A meta-analysis of ω-3 fatty acid supplementation studies conducted in patients with MDD found that only supplementation with pure EPA or fish oil mostly containing EPA demonstrated significant therapeutic effects [21]. In contrast, pure DHA or preparations containing mostly DHA did not show significant improvement in depression [21]. The lack of response to DHA supplementation may be due to the different metabolism of EPA and DHA in the brain and the preferential incorporation of DHA into cell membrane phospholipids, with DHA accounting for up to 40% of all fatty acids in some regions of the brain [33], and EPA mostly subjected to beta-oxidation [34]. However, both EPA and DHA may undergo conversion to SPM in the brain as 15-LOX is expressed by neurons and microglia [35]. In a mouse model of depression, intracranial administration of RvE1-3 or RvD1-2 was associated with reduced symptoms [36–38]. The contribution of EPA- and DHA-derived lipid mediators to clinical response in MDD therefore needs to be further investigated.

Efficacy of EPA supplementation was greater in the 4 g/d arm, consistent with findings from cardiovascular prevention studies [39] and suggests activation of the resolution phase of inflammation as a mechanism of clinical response. In the 4 g/d arm, plasma levels of both RvE2 and RvE3 were detectable in most responders, compare to no or low detection in non-responders. The strong association between changes in 18-HEPE and 15-HEPE, the precursors of RvE1-4, with changes in depression symptoms and plasma hs-CRP concentrations are highly suggestive of a mechanistic role of EPA-derived SPMs on inflammation and subsequent reduction in depression. A significant correlation between the increase in the concentration of selective SPMs and circulating immune cell characteristics following the administration of a high dose of a marine oil and SPM supplement was previously reported in another study [40].

A strength of this study was the selection of patients with MDD and with chronic inflammation, who are the ideal target for activation of the resolution of inflammation. In addition, we were able to correlate changes in plasma SPMs with hs-CRP, a marker of systemic inflammation, and with clinical symptoms of depression. One limitation of our study was the sample size, with a small number of subjects in each group of responders and non-responders. Our analyses were exploratory. The parent study was limited by a high placebo response rate [23]. In the original report, we remarked that placebo responders had lower baseline IDS-C30 scores than non-responders, a commonly reported finding [41], while clinical responders had similar mean baseline IDS-C30 scores compared to non-responders. Baseline plasma IL-6 levels in placebo responders versus non-responders also suggested that placebo may be more effective in subjects with MDD and lower baseline inflammation. This may impact the current findings. Our results strongly support a need for larger and definitive trials. We were also unable to report plasma concentrations of E-series and D-series resolvins at baseline and in the placebo arm due to levels below the detection limit of our assay.

Taken together, the results of our study indicate a dose-dependent reduction in depression symptoms following EPA supplementation. Among study participants receiving EPA, those who responded showed a greater ability to activate the synthesis of 18-HEPE. In addition, the increase in 18-HEPE following EPA supplementation in the 4 g/d arm was highly and negatively correlated with both systemic inflammation and symptoms of depression, highlighting the activation of the resolution of inflammation as a likely mechanism in the treatment of MDD.

## Supplementary information

Supplemental material

CONSORT Flowchart
