# Supplementary material for: Clinical response to EPA supplementation in patients with major depressive disorder is associated with higher plasma concentrations of pro-resolving lipid mediators
Source: Neuropsychopharmacology. 2023 Jan 12;48(6):929–35. doi: 10.1038/s41386-022-01527-7 (PMC10156711; doi:10.1038/s41386-022-01527-7)
Supplement: Supplementary file 1 — Supplemental material [file 41386_2022_1527_MOESM1_ESM.docx]

**Supplementary Table**. Baseline plasma levels of lipid mediators derived from EPA, DPA, DHA and AA, by treatment group.

|  | Placebo  (n=10) | EPA 1 g/d (n=13) | EPA 2 g/d (n=11) | EPA 4g/d (n=11) | P value* |
| --- | --- | --- | --- | --- | --- |
| **EPA** | | | | | |
| EPA, mol% | 0.55 (0.45) | 0.43 (0.31) | 0.48 (0.61) | 0.33 (0.30) | 0.20 |
| 5-HEPE, pg.mL | 84 (35) | 47 (30.) | 53 (25) | 61 (62.3) | 0.21 |
| 11-HEPE, pg.mL | 58 (29) | 53 (51) | 42 (37) | 54 (35) | 0.60 |
| 12-HEPE, pg.mL | 1216 (1311) | 761 (2040) | 1822 (3214) | 718 (420) | 0.60 |
| 15-HEPE, pg.mL | 108 (61) | 85 (54) | 105 (117) | 78 (52) | 0.78 |
| 18-HEPE, pg.mL | 62 (29) | 44 (18) | 50 (47) | 47 (25) | 0.57 |
| **DPA** | | | | | |
| DPA, mol% | 0.44 (0.24) | 0.37 (0.18) | 0.36 (0.23) | 0.32 (0.27) | 0.55 |
| RvD5__ DPA_, pg.mL | 15 (6) | 16 (4) | 15 (5) | 13 (3) | 0.62 |
| **DHA** | | | | | |
| DHA, mol% | 8.47 (4.17) | 7.94 (3.23) | 9.38 (5.30) | 6.77 (3.54) | 0.21 |
| 4-HDHA, pg.mL | 19 (9) | 63 (7) | 53 (35) | 58 (77) | 0.66 |
| 7-HDHA, pg.mL | 19 (12) | 19 (7) | 16 (20) | 26 (19) | 0.50 |
| 13-HDHA, pg.mL | 156 (288) | 198 (266) | 141 (171) | 117 (211) | 0.58 |
| 14-HDHA, pg.mL | 2997 (2116) | 2316 (3332) | 2859 (5316) | 1654 (947) | 0.97 |
| 17-HDHA, pg.mL | 27 (33) | 29 (26) | 32 (41) | 30 (12) | 0.83 |
| RvD1, pg.mL | 103 (65) | 80 (78) | 91 (135) | 111 (115) | 0.51 |
| **AA** | | | | | |
| AA, mol% | 11.21 (3.26) | 11.37 (4.68) | 9.39 (4.92) | 11.10 (5.91) | 0.96 |
| 5-HETE, pg.mL | 548 (560) | 409 (208) | 332 (118) | 602 (969) | 0.07 |
| 11-HETE, pg.mL | 1857 (1769) | 1871 (2461) | 1146 (1106) | 1647 (2472) | 0.26 |
| 12-HETE, pg.mL | 13201 (6704) | 8114 (22488) | 10568 (18417) | 8898 (7135) | 0.74 |
| 15-HETE, pg.mL | 1403 (360) | 1020 (923) | 1261 (1169) | 1265 (923) | 0.88 |
| PGE_2_, pg.mL | 812 (1858) | 1263 (2088) | 544 (966) | 1260 (2395) | 0.41 |
| TXB_2_, pg.mL | 1879 (2837) | 2542 (5316) | 1037 (3694) | 2295 (2694) | 0.38 |
| LTB_4_, pg.mL | 31 (21) | 39 (25) | 24 (14) | 21 (33) | 0.42 |

Data presented are median (IQR); * P value, Wilcoxon rank-sum test for continuous values

**Supplementary Figure.** Median changes in plasma EPA, DHA and AA from baseline to week 12 by treatment arm in responders and non-responders.
