# Supplementary material for: Clinical response to EPA supplementation in patients with major depressive disorder is associated with higher plasma concentrations of pro-resolving lipid mediators
Source: Neuropsychopharmacology. 2023 Jan 12;48(6):929–35. doi: 10.1038/s41386-022-01527-7 (PMC10156711; doi:10.1038/s41386-022-01527-7)
Supplement: Supplementary file 2 [file 41386_2022_1527_MOESM2_ESM.docx]

CONSORT Statement Flow Diagram

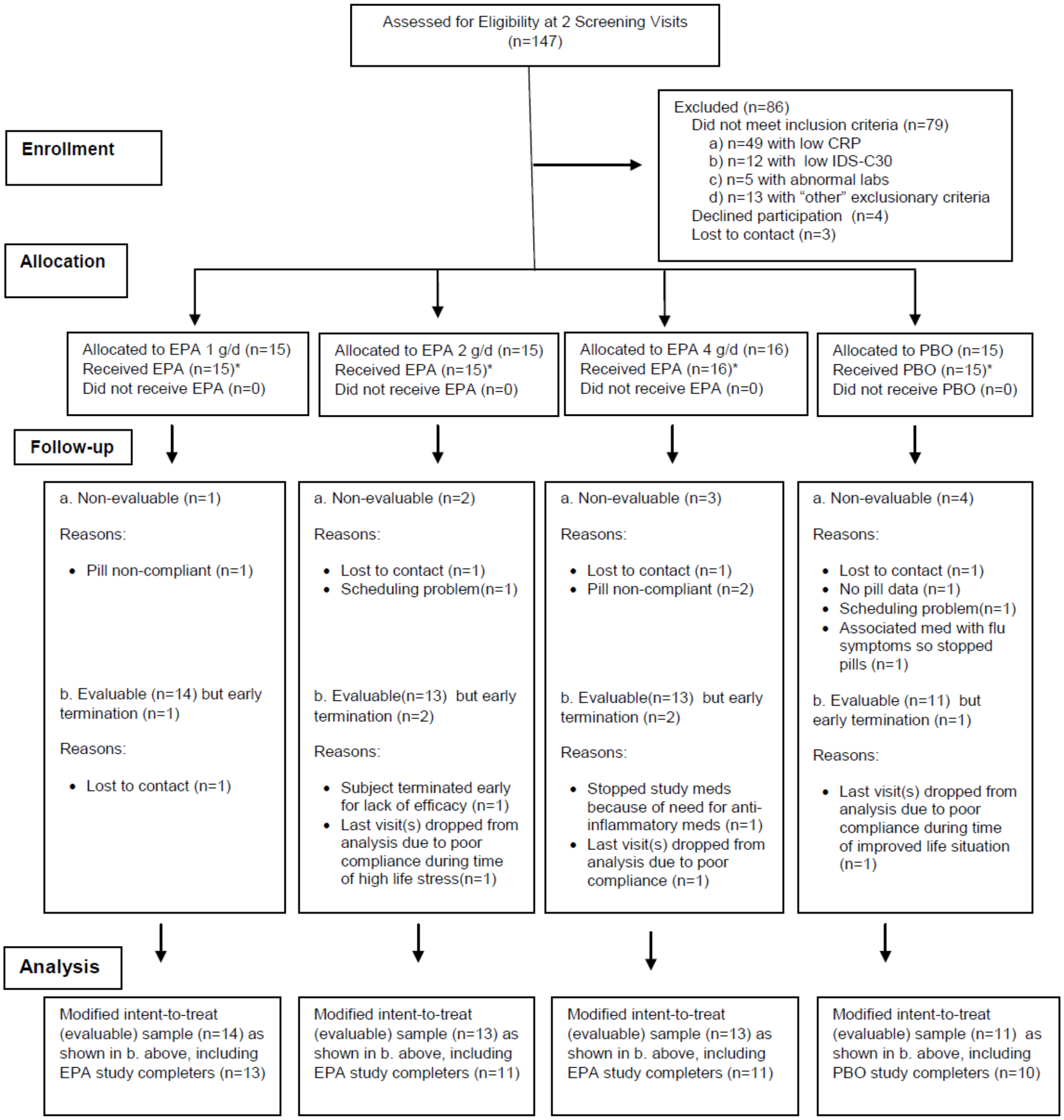

* Used for safety analysis
